# Plant–parasite coevolution: A weak signature of local adaptation between Peruvian *Globodera pallida* populations and wild potatoes

**DOI:** 10.1002/ece3.6248

**Published:** 2020-04-15

**Authors:** Camille Gautier, Sylvain Fournet, Christophe Piriou, Lionel Renault, Jean‐Claude Yvin, Eric Nguema‐Ona, Eric Grenier, Josselin Montarry

**Affiliations:** ^1^ IGEPP INRAE Agrocampus‐Ouest Université de Rennes 1 Le Rheu France; ^2^ Centre Mondial de l'Innovation‐Laboratoire de Nutrition Végétale Pôle Biocontrôle Groupe Roullier Saint‐Malo France

**Keywords:** cyst nematode, hatching, local adaptation, Peru, root exudates, wild *Solanum* species

## Abstract

Plant–parasite coevolution has generated much interest and studies to understand and manage diseases in agriculture. Such a reciprocal evolutionary process could lead to a pattern of local adaptation between plants and parasites. Based on the phylogeography of each partner, the present study tested the hypothesis of local adaptation between the potato cyst nematode *Globodera pallida* and wild potatoes in Peru. The measured fitness trait was the hatching of cysts which is induced by host root exudates. Using a cross‐hatching assay between 13 populations of *G. pallida* and root exudates from 12 wild potatoes, our results did not show a strong pattern of local adaptation of the parasite but the sympatric combinations induced better hatching of cysts than allopatric combinations, and there was a negative relationship between the hatching percentage and the geographical distance between nematode populations and wild potatoes. Moreover, a strong effect of the geographic origin of root exudates was found, with root exudates from south of Peru inducing better hatching than root exudates from north of Peru. These results could be useful to develop new biocontrol products or potato cultivars to limit damages caused by *G. pallida*.

## Graphical abstract

The present study tested the hypothesis of local adaptation between the potato cyst nematode *Globodera pallida* and wild potatoes in Peru, using a cross‐hatching assay between 13 populations of *G. pallida* and root exudates from 12 wild potatoes.

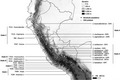

## 1 INTRODUCTION

Plants are constantly interacting with many organisms in their environment, and these interactions may be directly and/or indirectly beneficial or harmful for the plant (Bais, Weir, Perry, Gilroy, & Vivanco, 2006). On the one hand, beneficial associations could improve plant growth and health through better nutrient availability and protection against parasites (Berg, 2009; Mendes, Garbeva, & Raaijmakers, 2013; Richardson, Barea, McNeill, & Prigent‐Combaret, 2009). On the other hand, plants are attacked by a range of diverse parasites as bacteria, fungi, oomycetes, nematodes, or insects (Baetz & Martinoia, 2014). Parasites limit plant growth and/or reduce seed production. This leads to selection pressure on plants for resistance to parasites and in return on parasites to overcome host defences (Brown & Tellier, 2011). This intimate relationship between plants and parasites and the strong selection pressure that each exerts on the other results in a coevolutionary process (Burdon & Thrall, 2009; Gandon & Michalakis, 2002; Thompson, 1994).

By definition, coevolution is an evolutionary process that results from reciprocal selection and leads to adaptive genetic changes in organisms with a close and evident ecological relationship (Ehrlich & Raven, 1964; Janzen, 1980; Woolhouse, Webster, Domingo, Charlesworth, & Levin, 2002). In the framework of plant–parasite interactions and in a spatially structured environment, that is, disconnected parasite populations in different habitats, this coevolutionary process could lead to a pattern of local adaptation. Therefore, a given population of the parasite performs better in its own habitat (i.e., the host) than in another (the home versus away criterion of Kawecki & Ebert, 2004) and/or performs better in its own habitat than a population coming from a different one (the local versus foreign criterion of Kawecki & Ebert, 2004). Local adaptation has been addressed for several host–parasite interactions, and meta‐analyses showed (a) a general trend of local parasite adaptation, and that (b) the pattern of local adaptation is more frequent for traits related to parasite infection than for traits related to parasite multiplication (Greischar & Koskella, 2007; Hoeksema & Forde, 2008). According to Blanquart, Kaltz, Nuismer, and Gandon (2013), the representation of the true level of local adaption is better using the sympatric versus allopatric comparison when the number of sampled populations is high. Local adaptation is thus calculated as the difference between the relative fitness of population in its own habitat (sympatric combinations) and in other habitats (allopatric combinations) averaged over all populations, with no involvement made by confounding factors such as host or parasite effects.

For many soil‐borne parasites, plant infection does not occur without the release of stimuli by the host, used by the parasite to break dormancy of resting structures (sclerotia, oospores, chasmothecia, seeds, cysts) (Mendes et al., 2013). For instance, Balendres, Nichols, Tegg, and Wilson (2016) found that polar low‐molecular‐weight organic compounds in potato root exudates stimulated the germination of *Spongospora subterranean* resting spores. Also, Auger et al. (2012) showed that germination of *Phelipanche ramosa* seeds was triggered by stimulants derived from glucosinolates exuded by *Brassica napus* (oilseed rape). And for many cyst nematodes, root exudates from host plants are needed to stimulate the hatch and emergence of juveniles from the cyst (Perry, Moens, & Jones, 2018). In cyst nematode species, hatching is a trait related to the infection which is probably a good candidate trait to highlight local adaptation process as it is subject to a strong selective pressure for both partners of the interaction. Moreover, for spatially structured parasite species, such as nematodes which are characterized by limited active dispersal capabilities (Wallace, 1968), the parasite fitness was expected to decrease with the geographical distance between host and parasite populations (Adiba, Huet, & Kaltz, 2010; Hoeksema & Thompson, 2007; Kaltz, Gandon, Michalakis, & Shykoff, 1999).

Our case study involved the cyst nematode *Globodera pallida*, one of the major pest of potato crop worldwide (Oerke, Dehne, Schönbeck, & Weber, 2012; Turner & Evans, 1998) and listed in the EU Plant Health Directive 2000/29/EC also regulated by the European PCN Directive (2007/33/EC). This nematode originated from the Andean region of South‐America, the origin of its wild host potatoes and other *Solanum* species (Evans & Stone, 1977; Hijmans & Spooner, 2001). Its main range lies in Peru where Picard, Sempere, and Plantard (2007) highlighted five distinct genetic clades with a south‐to‐north pattern associated with a decreasing genetic diversity. *G. pallida* is a sedentary endoparasite. As other cyst nematode species, second‐stage juveniles (J2) hatch from the cyst after the perception of root exudates released by the host plant (Perry, 1986). Then juveniles penetrate inside the plant roots and induce a feeding site, the syncytium, which is an important nutrient sink for the plant (Jones & Northcote, 1972). Nematodes realize successive molts before becoming adult males and females. After mating, females die, their cuticle turns brown, hardens, and constitutes the cyst. While juveniles have low active dispersal capabilities, the passive dispersal of cysts could occur at longer distances. Using a hierarchical sampling strategy in Peru, Picard, Plantard, Scurrah, and Mugniery (2004) highlighted strong gene flow among *G. pallida* populations from the same region (the highest distance between fields being 35 km) and low gene flow between regions (the weakest distance between regions being 320 km). Accordingly, Picard and Plantard (2006) showed that nematodes sampled in fields located at <50 km apart could be considered as belonging to the same *G. pallida* population.

The hotspots of species richness for the host of *G. pallida*, the wild tuber‐bearing *Solanum* species, are in the Central and South‐American tropical highlands and more precisely in central Mexico, in north Argentina, Bolivia, Ecuador, and Peru (Hijmans & Spooner, 2001). According to Spooner and Castillo (1997), the phylogeny of wild potatoes is divided into four genetic clades and only clades 3 and 4 are present in Peru. Peru contains the highest number of species as well as the highest number of rare wild potatoes.

The strong dependence on the establishment and the perception of the chemical signal to hatch suggest a long coevolutionary history between *G. pallida* and *Solanum* spp. To explore the level of dependence between populations of *G. pallida* and root exudates from wild potato species, the present study addressed one main question: Is there a detectable pattern of local adaptation for the hatching trait between *G.* *pallida* populations and wild potatoes coming from Peru?

## 2 MATERIALS AND METHODS

### 2.1 Nematode populations

**FIGURE 1:**
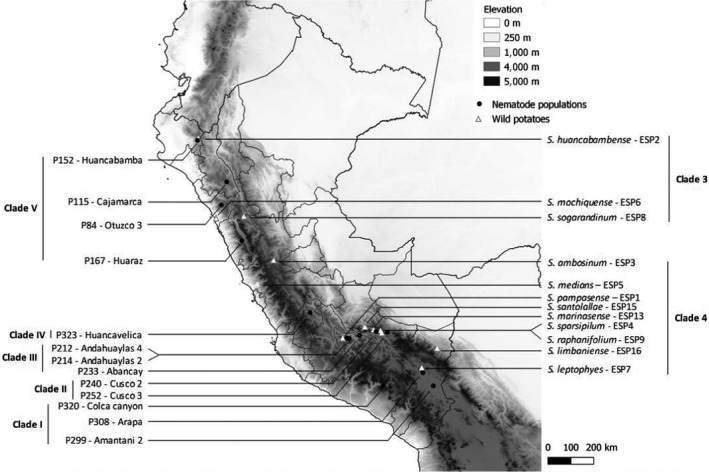
Map of Peru indicating the location of *Globodera pallida* populations and wild potatoes used in this study. Name, code, and clade membership of nematode populations are indicated on the left of the map, and name, code, and clade membership of wild potatoes are indicated on the right of the map

Thirteen populations of the potato cyst nematode *G. pallida*, originating from Peru and multiplied on potato cv. Désirée in a greenhouse, were used in this study. These *G. pallida* populations are members of the genetic clades described by Picard et al. (2007): clade I (P308—Arapa, P299—Amantani 2 and P320—Colca canyon), clade II (P240—Cusco 2 and P252—Cusco 3), clade III (P212—Andahuaylas 4, P214—Andahuaylas 2 and P233—Abancay), clade IV (P323—Huancavelica), and clade V (P152—Huancabamba, P84—Otuzco 3, P115—Cajamarca and P167—Huaraz) (Figure 1, Table S1A). Cysts were extracted from soil samples by a Kort elutriator and stored at 5°C.

### 2.2 Collection of root exudates

Twelve species of wild potatoes (one accession per species) from Peru were used to produce root exudates (Figure 1, Table S1B). For each accession, a pack of 50 seeds was provided by the Centre for Genetic Resources of the Netherlands (CGN). To break dormancy and stimulate sprouting, potato seeds were soaked for 24 hr in a solution of Ga3 at 700 ppm. Then, seeds were planted in 65:20:15 Irish peat/black peat/perlite in a greenhouse at 22/20°C day/night conditions with 16‐hr photoperiod. Three 3‐week‐old seedlings were transplanted in a pot and grown in 54:40:6 Irish peat/sand/clay in the same greenhouse, and four replicates (i.e., four independent pots) were done per species. Three weeks after transplanting the seeds, root exudates were collected on two occasions at 1‐week interval. For this, each pot was saturated and was leached twice with 100 ml of tap water, 30 min apart. Then, the leachate was filtered through a Whatman No. 1 filter paper. The leachate from all pots of the same species was pooled and stored at −20°C.

### 2.3 Hatching assays

The hatching assays were conducted in a climatic chamber at 18°C, in dark. For this, 12‐well plates (Costar^®^) were used and a sieve with 20 µm pores was added in each flat‐bottomed well. Each root exudate was carbon dosed using a FLASH 2000 CHNS/O Analysers (Thermo Scientific™) and standardized to 30 mg of carbon per gram of dry matter with autoclaved permuted water. Three cysts of each population with 1.5 ml of root exudates were put per sieve, and due to the number of available cysts, four to five replicates were realized per population and root exudates except for two populations (P252 and P320) with two replicates. Overall, to test the 156 comparisons (13 *G. pallida* populations * 12 potato exudates), 672 hatching assays were performed (i.e., on average 4.3 replicates per treatment). The number of hatched J2s was counted at days 2, 4, 10, 15, and 30 after the beginning of assays, and at each count, root exudates were replaced with fresh root exudates. At the end of the hatching experiment, cysts were crushed and the number of unhatched viable eggs was counted, in order to calculate a hatching percentage.

### 2.4 Data analysis

All statistical analyses were performed using the R software version 3.6. 1 (R Development Core Team, 2019). Normality of residuals and homogeneity of variances were checked by the Shapiro–Wilk and the Levene tests, respectively. When significant effects were detected, multiple comparisons of means were performed with the Tukey contrasts test (*α* = .05).

The effects of nematode populations, potato exudates, and their interaction on the final hatching percentage were tested through a multiway ANOVA. The geographic distance between nematode populations and wild potatoes ranged from 9.8 km to 1,570 km (Figure S1). Consequently, the geographic matrix, showing the distance between all pairs of nematode population—wild potato, was split in three categories following Adiba et al. (2010): sympatric (<225 km), near‐allopatric (between 225 and 900 km), and far‐allopatric (more than 900 km) (Figure S1). And the effect of those categories on the hatching percentage was tested through a one‐way ANOVA. Furthermore, a Pearson's product–moment correlation test was performed to study the correlation between the geographic distance (km), between nematode populations and wild potatoes, and the hatching percentage.

## 3 RESULTS

### 3.1 Nematode population and potato exudate effects

Regarding the final hatching percentage (at day 30) of 13 *G. pallida* populations confronted to 12 root exudates from wild potatoes, all coming from the south to the north of Peru, there were a significant exudate effect, a significant population effect and a significant effect of the interaction (Table 1).

**TABLE 1:** Results from the analysis of variance (ANOVA) assessing the effects of potato exudate, nematode population, and the corresponding two‐way interaction of these factors on the hatching of *Globodera pallida* juveniles

Source of variation	*df*	*F*‐value	*p*‐value
Exudate	11	113.3	<.0001
Population	12	35.1	<.0001
Exudate:Population	132	2.9	<.0001
Error	516		

**FIGURE 2:**
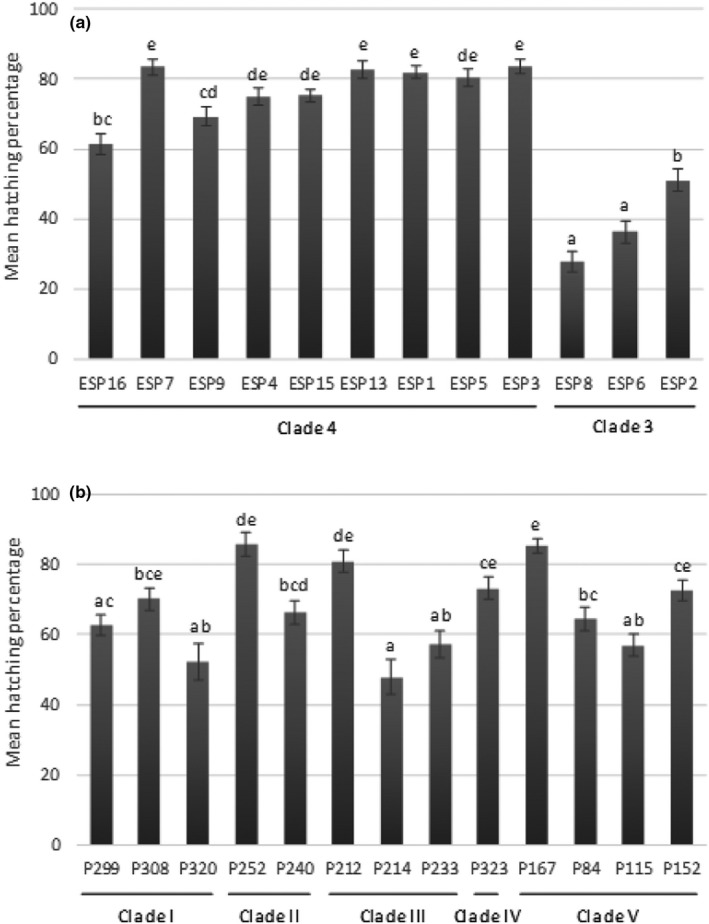
Mean hatching percentage of juveniles (mean values ± *SEM*) at the end of the experiment (D30) for (a) each root exudate from wild potatoes and for (b) each *Globodera pallida* population. The clade membership is indicated in the *x*‐axis. Letters represent homogenous groups identified by the Tukey contrasts test (*α* = .05)

The exudate effect was the highest (see *F*‐value in Table 1) and showed that the hatching was better for root exudates from clade 4 (accessions sampled in south and central Peru), than for root exudates from clade 3 (accessions sampled in north Peru) (Figure 2a). The Solanaceae which gave the best hatching percentage across all *G. pallida* populations was *Solanaum leptophyes* (ESP 3) with 83.58% whereas the lowest hatching was induced by *Solanum sogarandinum* (ESP 8) with 27.74%.

On the nematode side, the hatching percentage ranged from 47% (for population P214) to 86% (for population P167). The significant population effect (Table 1) did not match with the genetic clades of *G. pallida* (Figure 2b).

### 3.2 Local adaptation

**FIGURE 3:**
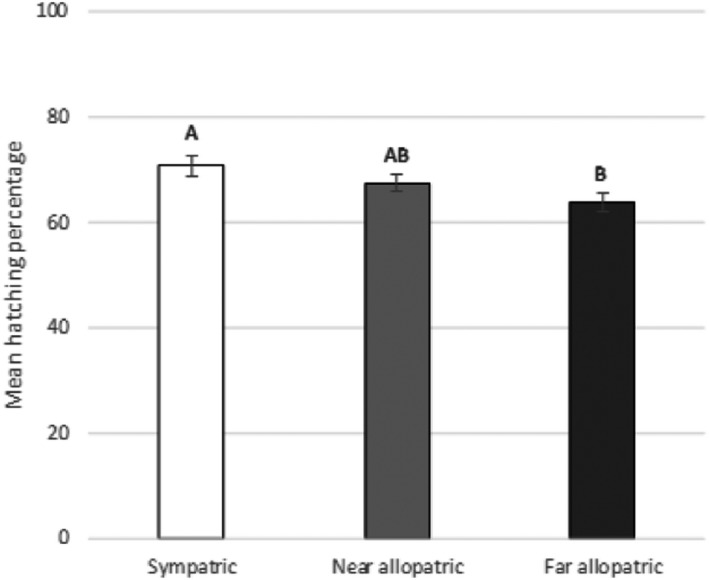
Mean hatching percentage of juveniles at day 30 (mean values ± *SEM*) for each combination defined according to the geographic distance between nematodes and potatoes, that is, sympatric, near‐allopatric and far‐allopatric. Letters represent homogenous groups identified by the Tukey contrasts test (*α* = .05)

To test the hypothesis of a local adaptation pattern, the hatching data matrix was divided into three categories based on the geographic distance between wild potatoes and *G. pallida* populations: (a) the sympatric combinations (<225 km), (b) the near‐allopatric (225–900 km), and (c) the far‐allopatric combinations (more than 900 km) (Figure S1). The ANOVA revealed a marginally significant effect (*F*_2,669_ = 2.99 and *p* = .051), but the Tukey post hoc comparison of means identified two groups: the sympatric combinations (mean ± *SEM* = 70.81 ± 1.94%) hatched better than the far‐allopatric combinations (mean ± *SEM* = 63.87 ± 1.86%), and the near‐allopatric combinations was intermediate (Figure 3).

### 3.3 Relationship between hatching percentage and geographic distance

**FIGURE 4:**
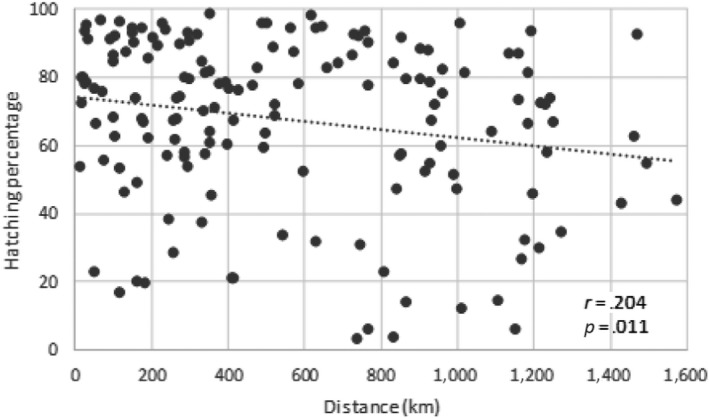
Relationship between the percentage of hatching and the geographical distance (km) between nematode populations and root exudates from wild potatoes

The relationship between the geographic distance, among wild potatoes and populations of *G. pallida*, and the hatching rate was negative (Figure 4). The correlation was weak (Pearson's cor coefficient = .204) but significant (*p* = .011), meaning that the hatching of J2 was better when populations of nematode and potatoes were geographically closed (Figure 4).

## 4 DISCUSSION

This experiment did not show a clear pattern of local adaption between the potato cyst nematode *Globodera pallida* and wild tuber‐bearing *Solanum* species for the hatching trait. In fact, splitting the data in three categories led only to a marginally significant effect, and we obtained the same result when the data were split in only two combinations (i.e., sympatric versus allopatric): the pattern of local adaption was marginally significant (*F*_1,670_ = 3.84 and *p* = .050) and the Tukey post hoc comparison of means identified an unique group (Figure S2). The absence of a strong signature of local adaptation between the nematode *G. pallida* and wild potatoes in Peru could result from the way we tested it and/or from biological characteristics of this nematode.

First, this result may be due to the use of *G. pallida* populations sampled in agricultural potato fields, whereas exudates were produced from potatoes coming from the wild compartment. Ideally, the best way to highlight a pattern of local adaptation would be to use *G. pallida* populations sampled directly on wild host plants. In that way, each partner of the interaction is geographically very close, leading to real sympatric (and allopatric) combinations.

Second, while low gene flow favors the maintenance of local adaptation patterns, strong gene flow could erase the signal of local adaptation (Kawecki & Ebert, 2004; Lenormand, 2002). In the case of cyst nematodes, the low active dispersal capabilities of free‐living stages in the soil (i.e., second‐stage juveniles and males), which may clearly favor local adaptation, may be strongly counteracted by the important gene flow among populations due to the passive dispersal of cysts at long distances. These gene flow were evidenced for the beet cyst nematode *Heterodera schachtii* (Plantard & Porte, 2004), for the carrot cyst nematode *H. carotae* (Esquibet et al., 2019), for the tobacco cyst nematode *Globodera tabacum* (Alenda, Montarry, & Grenier, 2014), and for the potato cyst nematodes *G. rostochiensis* (Blacket et al., 2019) and *G. pallida* (Picard et al., 2004). Alenda et al. (2014) showed a leading role of human activities in the passive dispersal of plant–parasitic nematodes.

Third, genetic drift is also expected to reduce local adaptation by reducing genetic diversity and by causing the random fixation of a reduced number of genotypes (Blanquart, Gandon, & Nuismer, 2012; Yeaman & Otto, 2011). The intensity of genetic drift is still unknown for many plant parasites, but two recent studies have estimated the effective population size in cyst nematodes. Using *H. schachtii* populations from the wild (Jan et al., 2016) or using artificial *G. pallida* populations (Montarry et al., 2019), both studies highlighted very low effective population sizes, resulting in a strong intensity of genetic drift in these species. Therefore, both characteristics, gene flow due to the passive dispersal of cysts and strong genetic drift, could lead to the weak pattern of local adaptation we reported here.

However, ours results, showing a nearly significant effect of sympatric versus allopatric(s) combinations and a negative significant relationship between the hatching of juveniles and the geographic distance between nematodes and potatoes, support the hypothesis of a coevolutionary history of *G. pallida* on wild potatoes. Based on our results, this hypothesis, which would be attested by further experiments using wild populations of *G. pallida*, seems more parsimonious than the alternative ones. Nevertheless, among the other candidate hosts of the Solanaceae family, wild tomatoes would be interesting to explore the hypothesis of a local adaptation with *G. pallida*. Indeed, tomato (*Solanum lycopersicum*) is also a good host of this nematode (Perry, Moens, & Jones, 2018), and their wild relatives are native to western South‐America (Peralta & Spooner, 2000), with a lot of these species being present in Peru (Peralta & Spooner, 2007).

Moreover, regarding the nematode population effect, there was no impact of *G. pallida* genetic clades (Picard et al., 2007) on the hatching of juveniles. This result could be due to a differential adaptation of each *G. pallida* population to contrasted local climate conditions (i.e., temperature, humidity). Hence, the greatest hatching for this species occurred between 13 and 25°C (Kaczmarek, Mackenzie, Kettle, & Biok, 2014) and the *G. pallida* populations used here were sampled from 2,980 m to 4,174 m along the Andean Cordillera in Peru with contrasted climate and geographic features (mountains, deep valleys). Thus, as hatching is a life‐history trait strongly depending on many factors but especially on temperature (see Perry et al., 2018 for a review), it is possible that our experimental running temperature (18°C) was far from the optimum temperature of each tested population. This was clearly shown for *H. schachtii* populations sampled in different environments (Fournet et al., 2018).

Conversely, a clear impact of the potato clades was observed with root exudates from the south of Peru (clade 4) inducing higher hatching of all *G. pallida* populations than root exudates from the north of Peru (clade 3). In the current context of the banishment of chemical nematicides, our results could be useful for potato breeding programs and for the establishment of new biocontrol products. First, wild tuber‐bearing *Solanum* of clade 4 could be preferred to develop products, based on root exudates, inducing the suicide hatch of juveniles in the absence of host plant (e.g., Devine & Jones, 2000; Lettice & Jones, 2015). Second, wild tuber‐bearing *Solanum* from clade 3 could be used by breeders for creating new potato cultivars, which would limit hatching of cysts and thus yield losses. A further study would be useful to confirm the impact of root exudates on European *G. pallida* populations, all derived from the north shore of the Lake Titicaca (Plantard et al., 2008). Additionally, a metabolomic approach would be interesting to understand in depth the differences of hatching efficiency of root exudates from clade 3 and clade 4.

## CONFLICT OF INTEREST

The authors declare that the research was conducted in the absence of any commercial or financial relationships that could be construed as a potential conflict of interest.

## AUTHOR CONTRIBUTION

**Camille Gautier:** Conceptualization (equal); Formal analysis (equal); Investigation (equal); Methodology (equal); Writing‐original draft (equal); Writing‐review & editing (equal). **Sylvain Fournet:** Conceptualization (equal); Funding acquisition (equal); Investigation (equal); Methodology (equal); Supervision (equal); Writing‐review & editing (equal). **Christophe Piriou:** Investigation (equal); Methodology (equal). **Lionel Renault:** Investigation (equal); Methodology (equal). **Jean‐Claude Yvin:** Conceptualization (equal); Funding acquisition (equal); Supervision (equal). **Eric Nguema‐Ona:** Conceptualization (equal); Funding acquisition (equal); Supervision (equal). **Eric Grenier:** Conceptualization (equal); Writing‐review & editing (equal). **Josselin Montarry:** Conceptualization (equal); Formal analysis (equal); Funding acquisition (equal); Investigation (equal); Supervision (equal); Writing‐original draft (equal); Writing‐review & editing (equal).

## Supporting information

Supplementary Material

## Data Availability

An excel file containing the raw data (including all replicates) of the cross‐hatching test, between 13 populations of *G. pallida* and root exudates from 12 wild potatoes, is available at data.inrae.fr (https://doi.org/10.15454/H1PJ5Q).
