# Supplementary material for: Plant–parasite coevolution: A weak signature of local adaptation between Peruvian Globodera pallida populations and wild potatoes
Source: Ecol Evol. 2020 Apr 15;10(9):4156–63. doi: 10.1002/ece3.6248 (PMC7244796; doi:10.1002/ece3.6248)
Supplement: Supplementary file 1 — Supplementary Material [file ECE3-10-4156-s001.docx]

**
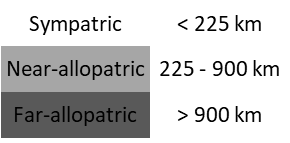

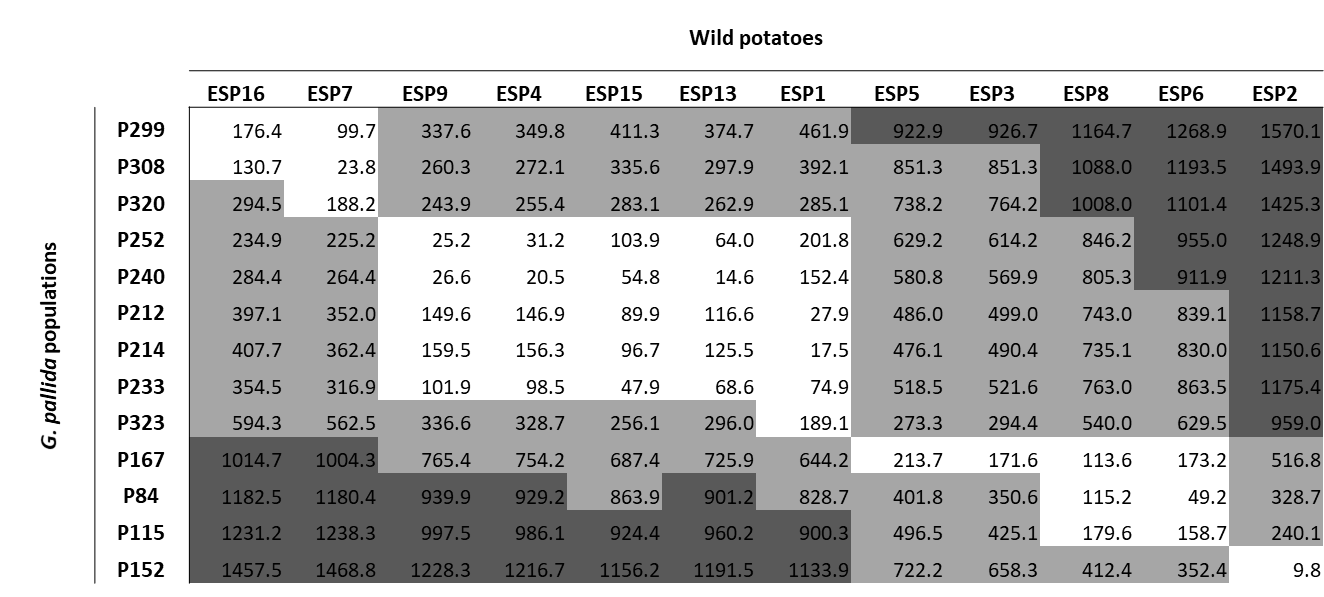
Supporting Information – Figure S1:** Matrix of geographical distances (km) between *G. pallida* populations and wild potatoes. The matrix was split into three combinations according to the geographical distance: sympatric in white, near-allopatric in light grey and far-allopatric in dark grey.

Note: Knowing that in Peru strong gene flow were highlighted between *G. pallida* populations located at less than 50 km apart (Picard & Plantard, 2006) and low gene flow among populations, from distinct Peruvian regions, located at 320 km apart (Picard et al, 2004), the first threshold (225 km) was chosen because it was contained between 50 and 320 km and because it was the lowest threshold allowing to defined at least one sympatric population for each potato exudate (e.g. ESP5). And the second threshold (900 km) was then defined to be at the middle between 225 km and 1570 km (the highest distance between a nematode population [P299] and a wild potato [ESP2]).

**Supporting Information – Tables S1A and S1B:** Summary tables of (A) wild potatoes and (B) *G. pallida* populations used in this study.

B

A

| **Wild potatoes** | **Genetic clade** | **Code** | **Longitude** | **Latitude** | **CGN accession number** |
| --- | --- | --- | --- | --- | --- |
| ***S. limbaniense*** | **4** | ESP 16 | 69°33'36.00"W | 14°4'48.00"S | 22720 |
| ***S. leptophyes*** | **4** | ESP 7 | 70°12'0.00"W | 14°54'0.00"S | 24374 |
| ***S. raphanifolium*** | **4** | ESP 9 | 71°50'59.99"W | 13°25'58.80"S | 17598 |
| ***S. sparsipilum*** | **4** | ESP 4 | 71°54'0.00"W | 13°19'58.80"S | 25177 |
| ***S. santollalae*** | **4** | ESP 15 | 72°32'59.99"W | 13°10'58.80"S | 23994 |
| ***S. marinasense*** | **4** | ESP 13 | 72°12'0.00"W | 13°18'0.00"S | 17594 |
| ***S. pampasense*** | **4** | ESP 1 | 73°28'1.19"W | 13°39'0.00"S | 17738 |
| ***S. medians*** | **4** | ESP 5 | 77°1'1.19"W | 11°22'1.20"S | 18307 |
| ***S. ambosinum*** | **4** | ESP 3 | 76°20'5.99"W | 10°22'17.40"S | 18322 |
| ***S. sogarandinum*** | **3** | ESP 8 | 77°34'22.79"W | 8°31'1.20"S | 23975 |
| ***S. mochiquense*** | **3** | ESP 6 | 78°45'0.00"W | 8°25'1.20"S | 20587 |
| ***S. huancabambense*** | **3** | ESP2 | 79°28'1.19"W | 5°13'58.80"S | 17719 |

| ***G. pallida* populations** | **Genetic clade** | **Code** | **Longitude** | **Latitude** |
| --- | --- | --- | --- | --- |
| **Amantani 2** | **I** | P299 | 69°42'50.64"W | 15°40'2.28"S |
| **Arapa** | **I** | P308 | 70°9'18.78"W | 15°6'30.12"S |
| **Colca canyon** | **I** | P320 | 71°46'51.96"W | 15°38'24.00"S |
| **Cusco 3** | **II** | P252 | 71°37'19.32"W | 13°24'37.68"S |
| **Cusco 2** | **II** | P240 | 72°5'19.98"W | 13°22'28.20"S |
| **Andahuaylas 4** | **III** | P212 | 73°12'35.94"W | 13°40'5.04"S |
| **Andahuaylas 2** | **III** | P214 | 73°18'32.04"W | 13°39'9.24"S |
| **Abancay** | **III** | P233 | 72°47'1.74"W | 13°32'20.64"S |
| **Huancavelica** | **IV** | P323 | 74°49'40.74"W | 12°34'31.68"S |
| **Huaraz** | **V** | P167 | 77°39'17.52"W | 9°32'29.10"S |
| **Otuzco 3** | **V** | P84 | 78°30'17.22"W | 8°2'28.14"S |
| **Cajamarca** | **V** | P115 | 78°17'2.82"W | 7°3'52.56"S |
| **Huancabamba** | **V** | P152 | 79°29'25.62"W | 5°19'4.98"S |

**
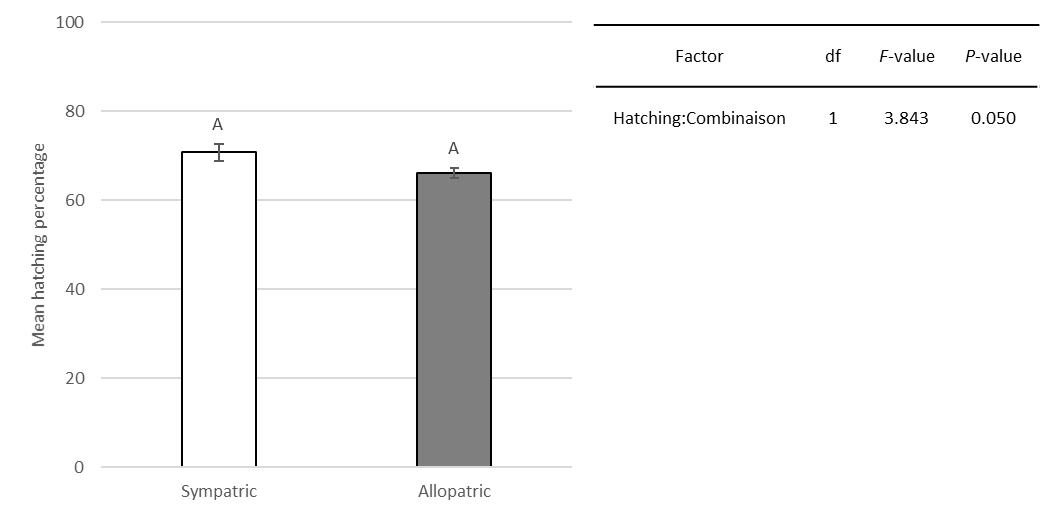
Supporting Information Figure S2:** Mean hatching percentage of juveniles at day 30 (mean values ± SEM) for each combination: sympatric and allopatric (which include near- and far-allopatric). Letters represent homogenous groups identified by the Tukey contrasts test (α = 0.05).
